# Advances in Endoscopic Ultrasound (EUS)-Guided Liver Biopsy

**DOI:** 10.3390/diagnostics13040784

**Published:** 2023-02-19

**Authors:** Daryl Ramai, Viraaj Pannu, Antonio Facciorusso, Banreet Dhindsa, Joseph Heaton, Andrew Ofosu, Saurabh Chandan, Marcello Maida, Barbara Lattanzi, Eduardo Rodriguez, Vicky H. Bhagat, Jayanta Samanta, Monique T. Barakat

**Affiliations:** 1 Division of Gastroenterology and Hepatology, University of Utah Health, Salt Lake City, UT 84132, USA; 2 Department of Medicine, Jersey Shore University Medical Center, Neptune City, NJ 07753, USA; 3 Section of Gastroenterology, Department of Medical Sciences, University of Foggia, 71100 Foggia, Italy; 4 Gastroenterology & Hepatology, University of Nebraska Medical Center, Omaha, NE 68198, USA; 5 Division of Digestive Diseases, University of Cincinnati, Cincinnati, OH 45219, USA; 6 Division of Gastroenterology & Hepatology, CHI Health Creighton University Medical Center, Omaha, NE 68124, USA; 7 Gastroenterology and Endoscopy Unit, S. Elia-Raimondi Hospital, 93100 Caltanissetta, Italy; 8 Endoscopy Unit, Hospital Sandro Pertini, 00157 Rome, Italy; 9 Division of Gastroenterology, Geisinger Commonwealth School of Medicine, Scranton, PA 18509, USA; 10 Department of Gastroenterology, Post Graduate Institute of Medical Education and Research, Chandigarh 160012, India; 11 Division of Gastroenterology, Stanford University, Stanford, CA 94305, USA

**Keywords:** endoscopic ultrasound, liver biopsy, endoscopy, percutaneous liver biopsy

## Abstract

Recent years have seen the emergence of endoscopic-ultrasound-guided liver biopsy (EUS-LB) as an effective alternative to traditional (percutaneous or transjugular) liver biopsy techniques. Comparative studies have demonstrated that both endoscopic and non-endoscopic approaches are similar in terms of diagnostic adequacy, accuracy, and adverse events; however, EUS-LB offers the advantage of reduced recovery time. Additionally, EUS-LB enables the sampling of both lobes of the liver as well as the advantage of portal pressure measurements. However, EUS-LB may be argued to have a high cost, although this procedure can be cost-effective if bundled with other endoscopic procedures. Approaches utilizing EUS-guided liver therapy, such as the administration of chemotherapeutic agents and EUS elastography, are in development, and their optimal integration into clinical care is likely to emerge in the coming years. In the present review, we evaluate the available literature on EUS-LB indications, contraindications, variations in needle biopsy techniques, comparative outcomes, advantages and disadvantages, and future trends and perspectives.

## 1. Introduction

A liver biopsy is a valuable tool to guide informed clinical decision-making regarding diagnosis and subsequent management. The first liver biopsy was performed by Dr Paul Elrich in 1883 in Germany [1]. Since then, there has been continuous growth and advancements in the methods and techniques used for procuring liver biopsy samples. The efficacy of the different techniques can be objectively assessed by the quality of tissue acquired. There are various metrics used to assess the adequacy of liver tissue samples. An adequate biopsy core sample is usually between 1.5 and 3 cm in length and 1.2 and 2 mm in diameter. The American Association for the Study of Liver Diseases (AASLD) states that an adequate sample size is 2–3 cm [2]. Furthermore, an AASLD statement indicates that fewer than 11 complete portal tracts (CPTs) may provide incorrect diagnosis, grading, or staging due to insufficient sample size [2].

## 2. Overview of Approaches for Liver Biopsy

### 2.1. Percutaneous Liver Biopsy (PCLB)

**Figure 1:**
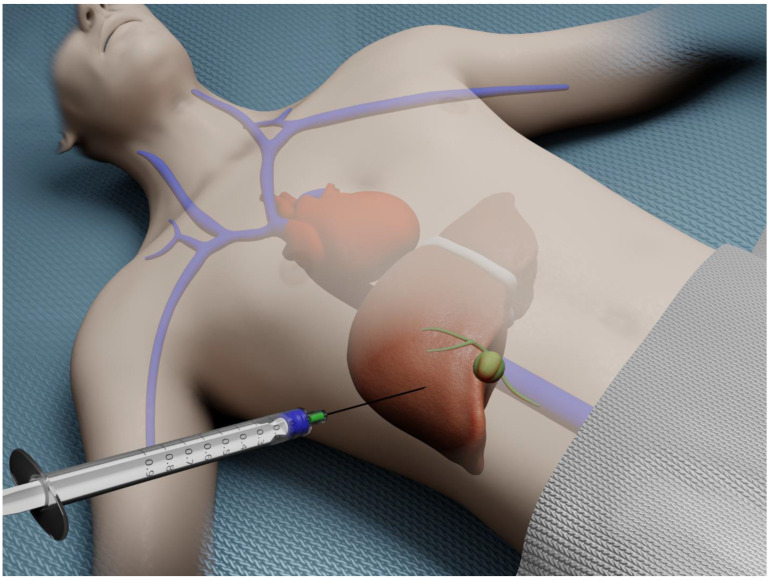
Illustration of bedside percutaneous liver biopsy.

Percutaneous liver biopsy (PCLB) is the most frequently employed modality for obtaining hepatic tissue. This approach is often considered to be technically the simplest technique for acquiring liver tissue. This method initially relied on a bedside percutaneous approach (Figure 1). 

However, ultrasound and CT imaging are often employed to ensure a more targeted and appropriate approach. The procedure is conducted under local anesthesia, followed by the insertion of a large bore (16–18 G) needle using surface landmarks to retrieve the required tissue sample. Though low-cost and reduced operator skill requirements are advantages to this approach, the disadvantages associated with percutaneous liver biopsies include increased sampling variability as well as the incidence of complications such as puncture site pain [3]. Additionally, this technique can be used to sample only the right liver. A prospective study conducted by Procopet et al. showed a higher degree of pain with PCLB vs. transjugular liver biopsies (TJLBs) seen by an increased need for intravenous analgesics (610 ± 580 mg of acetaminophen vs. 60 ± 280 mg; *p* < 0.005, respectively; 1.32 ± 3.55 mg of morphine vs. 0.30 ± 1.62 mg; *p* < 0.05, respectively) as well as fewer overall complications in the TJLB group in comparison to PCLB (15 patients vs. 26 patients; *p* = 0.002) while providing similar-quality tissue samples [3]. 

### 2.2. Transjugular Liver Biopsy (TJLB)

**Figure 2:**
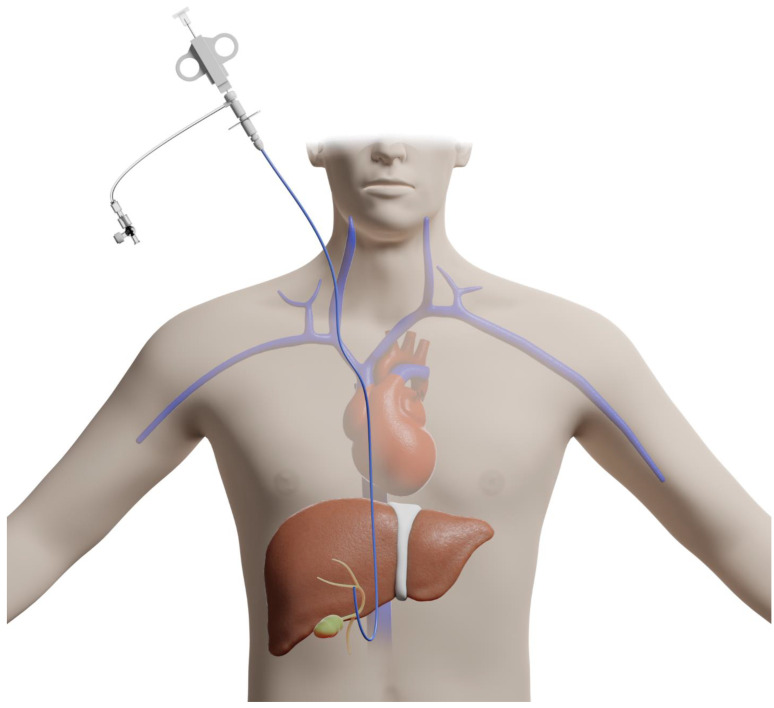
Illustration of a transjugular approach for liver biopsy.

The transjugular approach to liver biopsy involves accessing the jugular vein and advancing a guidewire down the inferior vena cava into the hepatic veins, thus, bypassing the liver capsule and peritoneum [4] (Figure 2). 

This approach provides an added advantage of portal hemodynamic monitoring while attaining tissue samples. This is beneficial in patients with advanced liver disease who require portal hemodynamic measurements for possible shunt placements [5]. Another advantage of this approach includes its applicability to patients with contraindications to PCLB, including coagulopathies, morbid obesity, or significant ascites. As mentioned above, there is a decreased incidence of post-procedure pain when compared to PCLB as the Glisson capsule is bypassed. Although samples collected by this approach are usually diagnostic, they are often found to be smaller and more fragmented [6]. The impact of fragmentation on diagnostic adequacy has not been formally studied; however, less fragmented samples tend to be preferred by pathologists.

### 2.3. Endoscopic-Ultrasound-Guided Liver Biopsy (EUS-LB)

**Figure 3:**
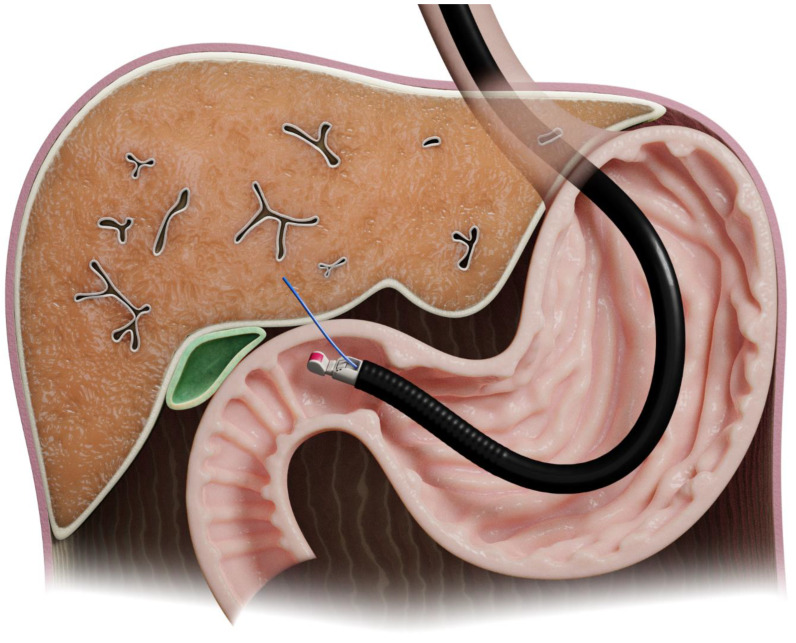
Illustration of endoscopic-ultrasound-guided liver biopsy.

The first study of EUS-LB in humans was in 2006 [7]. The left lobe can be approached using ultrasound guidance from the proximal stomach, while the right lobe can be accessed from the duodenal bulb [8] (Figure 3). 

A color doppler is employed to ensure there are no vascular structures along the needle path [9]. A meta-analysis showed that EUS-LB was associated with a 2.3% rate of adverse events with minor bleeding being the foremost complication [10]. EUS-LB offers the advantage of being able to sample multiple sites in the liver, which is difficult to obtain with both PCLB and TJLB. In a prospective study by Nguyen et al., it was seen that by employing EUS, small focal liver lesions not visualized by computer tomography (CT) scan were visualized by EUS [11]. Another study by Singh et al. involving 132 patients demonstrated the diagnostic superiority of EUS-FNA in detecting malignant hepatic lesions over CT scans (40 vs. 19, *p* = 0.008) [12]. 

## 3. Indications for Liver Biopsy

### 3.1. EUS-LB in Parenchymal Disease

EUS can effectively be used to assess liver parenchymal structure [13]. Tissue adequacy is assessed by CPTs and TSL. In a study by Stavropoulos et al., EUS-FNA with a 19 G needle extracted a median sample length of 36.9 mm with an average of nine CPTs, providing a 91% diagnostic adequacy and without any adverse events noted [14]. A clinical trial with 110 patients who underwent EUS-FNA, using 7–10 to-and-fro motions and the “fanning” technique provided a median aggregate length of 38 mm with a median of 14 CPTs, which was associated with 98% diagnostic accuracy. 

### 3.2. EUS-LB for Metastatic Diseases and Lesions

Most radiologic modalities are unable to detect very small lesions. A study by Awad et al. reported that EUS-FNA was able to detect lesions ranging from 0.3 cm to 14 cm in size [15]. Their study showed that EUS-FNA could detect lesions less than 0.5 cm in size in 28% of patients. Out of the malignancies detected, 41% of these were detected where CT scans were negative. EUS-FNA and associated results changed management in 86% of subjects [15]. A prospective study by Nguyen et al. found that EUS-LB was able to detect metastatic liver lesions in patients where preprocedural CT scans were only able to detect lesions in 21% of the study cohort [11]. EUS has also been found to be superior in determining the number of metastatic lesions compared to radiologic methods (40 vs. 19, *p* = 0.008) [12]. The aforementioned data attest to the fact that EUS-FNA holds great promise in both the accurate detection as well as diagnosis of metastatic lesions in the liver. 

### 3.3. EUS-LB for Hepatocellular Carcinoma

The ability of EUS-LB to detect and sample lesions in the liver that are too small for traditional imaging modalities is of critical importance, particularly in the selection of patients who are eligible for liver transplant or resection. Singh et al. assessed the accuracy of EUS and CT scans for detecting primary liver cancers in 17 patients with hepatitis B, C, and cirrhosis with a high risk of progression to primary hepatic malignancy [16]. The study reported that EUS had a diagnostic accuracy of 94%, which exceeded that of ultrasound (38%), CT scans (69%), and MRI (92%). In addition, EUS showed a significantly higher detection rate of nodular lesions in comparison with ultrasound (*p* = 0.03) and CT (0.002), as well as MRI (0.02) [16].

## 4. Endoscopic Technique and Needles

**Figure 4:**
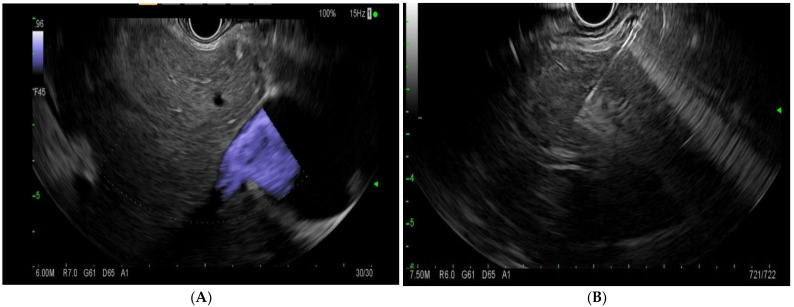
(**A**)—endoscopic ultrasound image of the liver with doppler. (**B**)—endoscopic-ultrasound-guided liver biopsy using a 19 G needle.

EUS-LB involves the use of an EUS linear echoendoscope and an endosonographic image with a doppler to guide the trajectory of the needle to obtain a liver biopsy (Figure 4). Prior to the procedure, doppler imaging is obtained to ensure vascular structures are present along the planned path of needle insertion [17]. This approach enables targeted sampling based on accurate endosonographic visualization to facilitate accurate sampling, diagnoses, and the subsequent management of various hepatic conditions. The first site to be identified is usually the left lobe of the liver, accessed from near the gastro-esophageal junction. Next, the right lobe of the liver is accessed from the duodenal bulb. The needle is passed at least 3 cm. In the event of wet heparinized suction, the stylet is primed with heparin and access is achieved. Suction is applied with a 20 cc vacuum syringe and completed with one pass utilizing up to five to-and-fro motions utilizing a fanning motion. Ideally, 2 such passes are made with a total of 10 actuations [17]. The specimen is promptly mounted on formalin. There have been various needle types and tissue acquisition techniques used in the assessment of hepatic parenchyma. Common needle types include 19 G flexible FNA needles (Expect; Boston Scientific, Marlborough, MA, USA); Franseen needles (Acquire; Boston Scientific, Marlborough, MA); reverse-bevel with tissue trap (ProCore; Cook Medical Inc, Winston-Salem, NC, USA); and 19 G Tru-Cut needles (Quick Core; Cook Medical Inc, Winston-Salem, NC). In the coming sections, we will review the most commonly used needle times for EUS-guided liver biopsy.

### 4.1. Tru-Cut Needles 

Tru-Cut needles often have a larger gauge and are applied with the expectation of gaining better tissue architecture and, subsequently, more adequate tissue samples. Tru-Cut needles were initially used with swine to obtain specimens from the spleen, liver, pancreas body, and left kidney [18]. Later, a human case series of nine patients was conducted by Gleeson et al. who showed that EUS of the left liver lobe with a 19 G Tru-Cut needle had a mean tissue sample length of 16.9 mm and a median of seven CPTs [19]. The use of Tru-Cut needles has been found to have more sample variability in comparison to alternative options. As an illustration of this, in a study by Dewitt et al. in which tissue samples were obtained from 21 patients, an accurate diagnosis was achieved in 90% of patients; however, most samples did not meet standard adequacy criteria for histologic assessment [13]. The inability to meet standard criteria for tissue diagnosis is believed to be partially due to the inflexible nature and inherent design limitation of the larger-sized Tru-Cut needle. Due to its inflexible design, it was deemed difficult to use, limiting its widespread adoption.

### 4.2. Fine Needle Aspiration Needles

EUS-FNA utilizes a 19 G needle to collect hepatic tissue. The first study to use FNA needles for acquiring hepatic tissue was by Stavropoulos and colleagues using a 19-gauge FNA needle [14]. They were able to achieve a median TSL of 36.9 mm (range 2–184.6 mm) and 9 CPTs (range 1–73 CPTs). A study by Diehl et al. in 2015 involving 110 participants, using a 19 G Expect (FNA) needle, saw a median tissue length of 38 mm with a CPT of 14, yielding an accurate tissue diagnosis of 98% [8]. The study reported no statistical difference in the yield between bilobar, left-lobe-only, or right-lobe-only biopsies. In this study, there was only one patient with an adverse event, which was bleeding in a patient with a pre-existing diagnosis of coagulopathy. Another study by Gor et al. showed that FNA liver biopsies had a tissue length of 14.4 mm with 9.2 CPTs yielding a diagnostic adequacy of 100% [20]. All biopsies were taken from the left lobe of the liver (via either a transgastric or transesophageal approach). Though FNA needles are widely applied, their tissue adequacy can have greater variability, depending on the needle size as well as operator experience.

### 4.3. Fine Needle Biopsy Needles

Biopsy needles have always been shown to outperform aspiration needles for tissue acquisition under EUS guidance. EUS-LB is no different. Multiple studies have shown higher rates of diagnostic tissue adequacy and diagnostic accuracy with FNB needles. A study by Sey et al. saw increased tissue adequacy and diagnostic yield with ProCore needles in comparison to 19 G Tru-Cut needles with a median length of 20 mm in comparison to 9 mm as well as more CPTs and fewer needle passes [21]. A retrospective study by Shah et al. with 24 participants using the 19 G SharkCore FNB needle achieved a diagnostic accuracy of 96% while having a median tissue length of 65.6 mm and 32.5 CPTs with a median of just two passes [22].

In a study by Bazerbachi et al., the diagnostic accuracy of EUS-LB, even with a smaller 22 G needle, was better than magnetic resonance elastography in detecting the early progression in NAFLD [23]. It also provided a median tissue sample length of 24 mm and a CPT of 26 while providing sample adequacy of 100% in the 41 patients studied. A prospective study by Ching-Companioni et al. saw that 19 G FNB needles provided longer specimen lengths and CPTS compared to 19 G FNA needles [24]. No severe adverse events (including bleeding) or differences in adverse event rates were reported between the two needles. Additionally, FNB needles require fewer passes to provide adequate tissue and accuracy when compared to FNA needles with statistically no significant difference in adverse events for sampling different lesion types (OR 0.96, 95%CI 0.37 to 2.54; *p* = 0.941) [25].

### 4.4. Various Tissue Acquisition Techniques

Various suction techniques can be applied while acquiring tissue samples through EUS for liver biopsy. Examples of common techniques include the dry suction technique (DRST), the wet suction technique (WEST), and the dry heparin technique. DRST involves the application of negative pressure through a dry syringe to acquire hepatic tissue. DRST increases tissue fragmentation and sample quality but increases the amount of blood in the tissue sample [26].

Wet suction has been developed to mitigate these issues by using a saline-flushed syringe instead of air. Mok et al. observed that the wet heparin technique provided increased tissue sample length with the least fragmentation and maximum CPTs when compared to the dry technique [27]. Wet suction techniques utilizing FNB needles were more accurate in diagnosing and staging NAFLD than magnetic resonance elastography [28]. A recent trial by Sharma et al. (BLOCS Trial) showed a possibly improved tissue acquisition with the wet suction technique with a total fragment length (46.5 mm vs. 34.5 mm, *p* < 0.0001), the length of the longest fragment (14 mm vs. 11 mm, *p* 0.0002), and the number of portal tracts (16 vs. 11.5, *p* = 0.0006) [29]. Similar results were seen with other lesions [30].

## 5. Contraindications

It is imperative to be aware of the various relative and absolute contraindications associated with EUS-LB in order to minimize risks. The precautions and contraindications of EUS-LB are the same as those for percutaneous liver biopsy [2]. Firstly, EUS-LB should only be performed on adequately sedated and cooperative patients with the appropriate monitoring of heart rate, blood pressure, oxygen saturation, and telemetry. As with any procedure, clinical judgment on the benefit of the procedure and its results on actual disease management should be considered. Patients in anticoagulation and antiplatelet therapy also require further consideration and an assessment of the relative safety of EUS-LB. AASLD guidelines generally recommend the discontinuation of antiplatelet medications 10 days prior to liver biopsy and can be restarted 48–72 h after the procedure. Anticoagulation with heparin and related products is generally advised to be held 12–24 h prior to the procedure, while warfarin should be held at least 5 days prior to the procedure [2]. Traditionally, large-volume ascites have been thought to be a contraindication to liver biopsy. Altered anatomy (e.g., Roux-en-Y gastric bypass) is not an absolute contraindication because a left lobe liver biopsy can still be achieved even in patients with partial gastric resection [31].

## 6. EUS-LB0-Associated Outcomes

A meta-analysis by Baran et al., which included 23 studies and a total of 1488 liver parenchymal biopsies from 1326 patients, showed a pooled diagnostic yield of 93% (1336/1436), which was comparable to percutaneous liver biopsies [32]. After excluding the studies using the QuickCore needle (no longer available in the market), the diagnostic yield rose to 95%. Between the various needle types, it was found that EUS-FNB needles were superior to EUS-Tru-Cut biopsy needles with a yield of 95% (1271/1332) vs. 63% (65/104), respectively (*p*-value < 0.001). The diagnostic yield was found to be better with a bi-lobar approach (99.7%, 704/706) vs. single (right or left)-lobar approach (91.4%, 544/595) (*p*-value < 0.001). These findings indicate an improved diagnostic yield as well as tissue acquisition with 22 G Acquire (Franseen) FNB needles when compared to 19 G FNA needles.

Amongst FNB needles, the Franseen needle (Acquire) has shown superior tissue adequacy and diagnostic yield. Its three symmetric cutting edges are designed to get deep into the tissue and obtain ample tissue volume. A study by Aggarwal et al. saw an improved diagnostic yield from Franseen-tip (Acquire) needles vs. Fork-tip (SharkCore) needle systems at 97.2% vs. 79.4%, respectively (*p*-value < 0.001) [33]. All liver biopsies were performed on the left lobe. In a meta-analysis by Baran et al., a comparison between Fork-tip vs. Franseen-tip FNB needles showed that tissue length was found to be significantly greater in Franseen-tip needles, with 39.3 ± 6.8 mm vs. 75.9 ± 9.9 mm (*p*-value = 0.002) [32]. Baran et al. also assessed the use of suction in tissue acquisition and found a statistically significant improvement in CPTs acquired with 14.6 ± 1.7 vs. 30 ± 4.1; *p*-value < 0.001 when suction was applied compared to the slow-pull technique [32]. No statistically significant difference in TSL was observed.

A study by Nieto et al. assessed the efficacy and adequacy of samples collected by a modified single-pass wet suction tissue collection technique and found them to be comparable to other techniques [34]. Both lobes of the liver were sampled in this study and showed a median maximum intact core tissue length of 2.4 cm (IQR, 1.8–3.5), a median total specimen length of 6 cm (IQR, 4.3–8), and a median number of complete portal tracts (CPTs) per TSL of 18 (IQR, 13- 24). The mean number of CPTs per sample length was 7.5 cm. A study by Ching et al. further showed a benefit in a single-pass approach but with three actuations in comparison to a single actuation [35]. Compared with 1:1 (pass: actuation), biopsy sampling with 1:3 yielded more CPTs (mean (standard deviation), 17.25 (6.2) vs. 24.5 (9.88); *p* < 0.008) and a longer aggregate specimen length (6.89 cm (1.86) vs. 12.85 cm (4.02); *p* < 0.001). Further, the study reported comparable outcomes between left and right lobe biopsies (in the right lobe only, CPT counts were ≥ 11 in only 20% of the 1:1 group and 65% in the 1:3 group while for left lobe biopsy, 25% of patients had CPT count ≥ 11 in the 1:1 group compared with 60% in the 1:3 group). Both these studies utilized 19-gauge FNB needles.

Given the current data, a 19-gauge FNB is preferred over a 19-gauge FNA needle, with better LB specimens. If a 19-gauge FNB needle is not available, a 19-gauge FNA needle should be adequate, although it works best if used with the “wet suction” method [31].

## 7. Economics of EUS-LB

There have not been many studies conducted to assess the exact cost-effectiveness of EUS-guided liver biopsies. A review by Masson et al. attempted to assess the cost-effectiveness of EUS-LB against current interventions of choice in patients with non-alcoholic fatty liver disease from data from the Center for Medicare Services and from the National Inpatient Sample Database [36]. Factors impacting the cost included the cost of deeper sedation requirements for EUS-LB and improved diagnostic yield and adverse events associated with EUS-LB. In their review, they found a similar cost between EUS-LB and percutaneous liver biopsy at USD 2610 vs. USD 2660 and QALY (quality-adjusted life years) of 3.8741 vs. 3.8738 years lived in perfect health. In addition, in patients receiving an EGD as well as an EUS-LB, it can prove to be even more cost-effective with concomitant indications for both. EUS-LB can also reduce the need for further testing with improved histologic variability as both lobes of the liver can be accessed with EUS [37].

## 8. Advantages and Disadvantages of EUS-LB

### 8.1. Procedure Duration and Pain

EUS-guided liver biopsy is a relatively short procedure; however, PCLB likely offers even shorter procedure times. Facciorusso et al. reported in their study a mean duration of 7 min (range, 5–11) for EUS-LB versus PCLB and 1 min (range, 1–3) for PCLB, *p* < 0.001, with no evidence of adverse events [38]. A randomized controlled trial by Bang et al. reported a mean procedure duration of 6.5 (±4.0) mins for EUS-LB versus 2.2 (±1.7) mins for PCLB [39]. While there are sparse data comparing procedure times for EUS-LB and TJLB, the literature reports a range of 15–48 min for TJLB [40].

A tertiary center retrospective study reported that EUS-LB was associated with shorter recovery times when compared to other liver biopsy methods (EUS-LB, 90.8 ± 52.2 min; PCLB, 235.5 ± 35.9 min; and TJLB, 141.3 ± 49.7 min) [41]. Typically, after PC-LB, patients are placed in the right decubitus position to tamponade the puncture site for about 2–4 h (depending on the institutional protocol). This contrasts with EUS-LB, where there are no positional requirements or restrictions prior to hospital discharge [42].

In a case-matched study, comparing 1 h recovery vs. 2 h recovery found that 2 h recovery showed no advantage in 90% of the cases with the remaining 10% requiring more observation due to postprocedural pain [43]. Current AASLD guidelines for observation post-procedure for traditional liver biopsies is 2–4 h [2]. With the reduced duration of stays for post-procedure observation for EUS-LB, it has the potential for a faster turnaround time.

### 8.2. Accuracy in Comparison to Other LB Modalities

Pineda et al. conducted a study assessing the diagnostic adequacy between EUS-LB, PCLB, and TJLB [37]. They found that tissue samples from EUS-LB where both hepatic lobes were accessed had significantly greater TSL with a median of 40 mm (TSL range: 30 mm–82 mm) compared to PCLB with a median TSL of 25 mm (TSL range: 15 mm–38 mm) with a *p*-value of < 0.001. CPTs were also noted to be significantly greater in the bi-lobed EUS-LB sample with a median CPT of 17 (CPT range: 10.5–29 CPTs) in comparison to PCLB having a median of 10 CPTs (CPT range: 7–16 CPTs) with a *p*-value of < 0.0006. In the same study, the authors found significantly greater TSL in the EUS-LB group, even in comparison to the TJLB group with a median TSL of 40 mm (TSL range: 30 mm–82 mm) vs. 34 mm (TSL range: 24 mm–48 mm) with a *p*-value of 0.01. However, the number of CPTs between these two groups was not found to be statistically different. A two-center study by Facciorusso et al. involving 116 patients compared the diagnostic yield between PCLB [38]. They found PCLB and EUS-LB to have comparable diagnostic yields. The only statistically significant difference found was in median TSL at 27.4 mm (IQR, 21 mm–29 mm) vs. 18.5 mm (IQR, 10.1 mm–22.4 mm) with a *p*-value of 0.02. They also found EUS-LB to be a significantly longer procedure (7 min, 5–11 versus 1 min, 1–3 of PC-LB; *p* < 0.001).

A meta-analysis involving seven studies showed similar findings in addition to finding no statistically significant difference in the median TSL between PCLB and EUS-LB [44]. A study by Ang et al. assessing the yield of histologic cores between FNA and FNB needles saw a significant difference between 19 G FNA needles when compared to 22 G Acquire FNB needles (67.4% (29/43) vs. 94.1% (16/17); *p*-value = 0.032) [45].

### 8.3. Complication Rates between LB Modalities

A meta-analysis conducted by McCarty et al. assessed the incidence of adverse events amongst EUS-LB, PCLB, and TJLB [46]. The meta-analysis included five studies comparing all three modalities and found that there was no statistically significant difference among adverse events between EUS-LB vs. PCLB and EUS-LB vs. TJLB. A study by Abdelfattah et al. compared the incidence of post-procedure pain in EUS-LB patients and PCLB patients. The EUS-LB group had 65 patients enrolled and PCLB had 152 [47]. In the study, 1 patient in the EUS-LB group had increased pain and required analgesics, whereas 12 PCLB patients had increased pain with 8 of them requiring analgesics; however, these findings were not found to be statistically significant. A randomized trial by Bang et el. reported that pain scores using the Visual Analog Scale (VAS) were significantly higher for the PCLB group at 1 hour post-procedure (mean change in VAS 2.0 vs. 0.43, *p* = 0.038), but there was no significant difference in pain score changes between the cohorts at post-procedure days 1, 7, and 30 [39]. The study reported no adverse events in either cohort. Larger long-term studies are warranted to further assess differences in complication rates between EUS-LB, PCLB as well as TJLB.

## 9. Overview of Present State and Future Uses of EUS-LB

Recent years have seen the emergence of endoscopic ultrasound liver biopsy (EUS-LB) as an effective alternative to traditional (percutaneous or transjugular) liver biopsy techniques. Comparative studies have demonstrated that both methods are similar in terms of diagnostic adequacy, accuracy, and adverse events; however, endoscopy offers the advantage of reduced recovery time. While peritoneal dissemination with EUS-LB has not been reported, endoscopists and patients should be aware that there may be a risk.

EUS-LB enables the sampling of both lobes of the liver. To this end, refinements in endoscopic needles have improved the diagnostic yield of EUS-LB. The role of hospital or institution volume may play a role in sampling adequacy and the need for re-biopsy. Huang et al. showed that high facility volume was associated with decreased rates of technical failures (as assessed by the need for re-biopsy) following EUS sampling procedures [48]. However, large studies are needed to understand the relationship between hospital volume status and outcomes associated with EUS-LB.

Other diagnostic models developed for EUS have the potential to augment, as well as potentiate, the diagnostic applicability of EUS. One such use is EUS-guided portal pressure gradient measurements. Other methods that can augment liver tissue acquisition include EUS-elastography to assess liver tissue firmness and further characterize focal as well as hepatic parenchymal diseases. A study by Sandulescu et al. showed that real-time elastography had a sensitivity of 92.5%, a specificity of 88.8%, and an accuracy of 88.6% [49]. Contrast-enhanced EUS with the use of ultrasound-enhancing contrast agents acts as a possible method to improve lesion characterization. A study by Minaga et al. showed that the use of contrast agents improved the accuracy of detection of metastatic lesions in the left lobe of the liver up to 98.4% while compared to EUS alone at 93.4% and CT scan at 90.6% [50].

A variety of new tools and techniques have also been developed for EUS to increase its diagnostic potential in a variety of conditions. Apart from just obtaining tissue samples, EUS can be used for therapeutic modalities such as injecting ethanol in primary or metastatic lesions in the liver [51]. Other therapeutic modalities being employed include the use of brachytherapy and the use of both photodynamic and thermoablative procedures.

A study by Faigel et al. using porcine models attempted to observe the pharmacokinetics of chemotherapeutic medications injected into portal circulation by EUS [52]. When observed for doxorubicin, the investigators found a 5-fold increase in hepatic levels (35,450 vs. 6930 ng/g) and a 30-fold decrease in cardiac levels (153 vs. 4805 ng/g) compared with systemic administration, both with a *p*-value < 0.05. This shows promise for targeted delivery for hepatic metastatic or primary malignant lesions while possibly reducing adverse systemic effects; however, more research with randomized trials in humans is required to validate these models.

Overall, EUS-LB is a procedure that has shown tremendous advancements since its inception and shows growing potential with improved tissue adequacy and diagnostic accuracy, as well as economic viability. Approaches for EUS-guided, liver-directed diagnosis and therapy, such as the administration of chemotherapy agents, EUS elastography, and portal pressure gradient measurements, are in development, and their optimal integration into clinical care is likely to emerge in the coming years.

## Data Availability

Not applicable.
